# The Development and Application of Novel IR and NMR-Based Model for the Evaluation of Carminative Effect of *Artemisia judaica* L. Essential Oil

**DOI:** 10.1155/2014/627038

**Published:** 2014-12-29

**Authors:** Muhammed Alzweiri, Ibrahim M. Alrawashdeh, Sanaa K. Bardaweel

**Affiliations:** ^1^Department of Pharmaceutical Sciences, Faculty of Pharmacy, The University of Jordan, Amman 11942, Jordan; ^2^Department of Biological Sciences, Faculty of Sciences, Al-Hussein Bin Talal University, Ma'an 20, Jordan

## Abstract

*Artemisia judaica* L. is a medicinal plant that is traditionally used to relieve abdominal pains through its carminative activity. In this study, spectroscopic analysis was employed to investigate the carminative activity associated with *A. judaica*. Using infrared spectroscopy, the carminative activity was evaluated based on the first derivative of IR-characteristic stretching signal of CO_2_. Our results indicate that *A. judaica* oil effectively reduced the response of CO_2_ signal equivalent to thymol standard. Additionally, ^1^H-NMR spectroscopy was utilized to assess surface activity of *A. judaica* crude oil through the reduction of interfacial tension in a D_2_O/CDCl_3_ system. Apparently, 10 mg of the oil was able to solubilize water in a chloroform layer up to 4.3% (w/w). In order to correlate the observed surface activity of the oil to its actual composition, GC-MS and GC-FID structural analysis were undertaken. The results revealed that the oil composition consists of oxygenated terpenes which might be responsible for the carminative effect. Furthermore, owing to its sensitivity, our model provides a fundamental basis for the pharmacological assessment of trace amounts of oils with high precision and accuracy.

## 1. Introduction

The large genus* Artemisia* L. (Fam. Asteraceae) encompasses distinctive medicinal plants which are known for their biological and chemical diversity [1, 2].

*Artemisia* species are found in many traditional preparations and are frequently used for the treatment of diseases such as malaria, hepatitis, cancer, inflammation, and infections by fungi, bacteria, and viruses [3]. Additionally,* Artemisia* species are commonly used for their antispasmodic, carminative, and anthelmintic properties [3–6].

*Artemisia judaica* is a perennial fragrant shrub that grows broadly in North African and Middle-Eastern countries. Distinctive ethnopharmacological utilization of* A. judaica* (wormwood) for the relief of abdominal pains has been reported in regions where* A. judaica* is inhabitant [7–10]. In addition, the isolated active ingredients from* A. judaica* demonstrated antibacterial, antifungal, and cytotoxic activities [1, 11–13].* A. judaica* contains artemisinic acid, methyl wormwood, artemisinic alcohol, and other essential oils, particularly eucalyptol, artemisia ketone, camphor, caryophyllene, and piperitone [2, 14]. Interestingly, it has been suggested that the characteristic composition of its essential oil endorsed* Artemisia* species such as assorted biological activity [3–6].

Essential oils have been commonly employed for their antiseptic and medicinal properties. Furthermore, essential oils have been utilized for the relief of abdominal pains [15–17]. Few studies reported receptor-medicated mechanism for the spasmolytic effect of essential oils [18–21] while others speculated non-receptor-mediated mechanism [22–24]. H. G. Grigoleit and P. Grigoleit reported that glucuranated metabolites of menthol and their continuous enterohepatic circulation are responsible for calcium channel antagonism and consequently the muscular relaxation effect of menthol [25]. Additionally, disturbance of the membrane integrity in enterocytes, resulting from the surface activity of the oil, has been proposed as a mechanism underlying the muscular relaxation associated with various essential oils [24, 26, 27].

Owing to the complexity of essential oils' composition and their prompt activity against abdominal pains, there is a strong belief that the activity of essential oils in treatment of abdominal pains is due to a non-receptor-mediated mechanism [28, 29].

Surface tension between water and gas bubbles is usually attributed to abdominal pains [30, 31]. Thus, the reduction of surface tension, caused by the surface activity of essential oils, has been suggested to relieve abdominal pains [30, 31]. The surface activity of the oil enhances coalescence of small bubbles and consequently their removal from the gut [32–34]. Evidently, pure thymol demonstrated remarkable activity against surface tension [22, 35]. Due to its ionisable phenolic group, the hydrophilic-hydrophobic balance of thymol, confers it the highest known surface activity among essential oils [23, 24].

Evaluation of the oil's surface activity is usually measured by special apparatus that requires special attention and excessive handling skills, such as the apparatus developed by Harries et al. to measure the antifoaming activity of certain essential oils [23, 31]. Additionally, surface activity evaluation of oils through the measurement of critical micelle concentration and disturbance of phospholipid particles' integrity were also reported [22, 24]. Poor precision, insufficient accuracy, and the requirement of relatively large amount of the essential oils are usually the major drawbacks associated with the reported methods [24]. Besides, the reported methods suit pure oil more than the crude oil extracts as they rely on numerical values of pure materials, such as molar absorptivity, surface pressure, and critical micelle concentration values [23, 24].

In this study, a novel model relying on sample-conservative spectroscopic methods, including infrared (IR) and nuclear magnetic resonance (NMR) [36–38], was used to evaluate the interfacial tension reduction and the antifoaming activities of* A. judaica* essential oil. In contrast to the previously reported models, our proposed nondestructive analysis requires diminutive amount of oil samples. Noticeably, both reproducibility and accuracy are distinguished advantages of our proposed IR and NMR-based model [39–41].

## 2. Materials and Methods

### 2.1. Chemical and Reagents

Deuterated water (D_2_O) 99.9% and deuterated chloroform (CDCl_3_) 99.8% containing 1% v/v trimethylsilane (TMS) were purchased from Sigma-Aldrich, USA. Methanol (analytical grade) was purchased from Loba Chemie, India. Thymol was used as positive control and purchased from Prolabo, UK, while paraffin oil was used as negative control and purchased from Sigma-Aldrich, USA. Hexane 95% GC grade was purchased from Tedia, USA.

### 2.2. IR, NMR, GC-MS, and GC-FID Instrumentations

IR analysis was undertaken by attenuated total reflectance-Fourier transformed-infrared spectrometer (ATR/FT-IR) with portable design from Bruker under the name of alpha ECD-ATR instrument.

^1^H-NMR data were acquired on a Varian Oxford-300 NMR spectrometer operating at a proton resonance frequency of 300 MHz controlled by VnmrJ 2.2D software.

GC-MS was used for identification and quantification of essential oils. GC-MS analysis was performed on a ThermoQuest gas chromatograph coupled to mass spectrometer (QP2010) equipped with Rtx-5MS polar capillary column stationary phases. 1 *μ*L of 0.1% oil solution (in n-hexane dried over anhydrous Na_2_SO_4_) was injected into a TRACE GC 2000 Series (ThermoQuest CE instruments, Austin, TX, USA) gas chromatography equipped with a split/splitless injector. Split ratios of 1 : 30 for diluted and 1 : 100 for undiluted oil samples were used. The column was an Rtx-5MS fused silica capillary column (30 m × 0.25 mm and 0.25 *μ*m film thickness) consisting of cross bond (5% diphenyl and 95% dimethyl polysiloxane). Helium (He) was used as a carrier gas at a flow rate of 1.0 mL/min. The GC was interfaced with a GCQ plus (ThermoQuest, Finnigan) mass detector operating in the electron ionization (EI) mode (70 eV). The mass spectra were generally recorded over 40 to 650 amu full-scan mode that revealed the total ion current (TIC) chromatograms. A linear temperature program was adapted to separate the different oil components as follows: Initially, the column was maintained at 70°C for 1 min, ramped at 5°C min^−1^ to 130°C, and maintained for 10 min; a second ramp was then applied at 8°C min^−1^ to final temperature of 210°C which was held isothermally for 2 min. The temperatures of the injector base, transfer line, and ion source were maintained at 250, 250, and 200°C, respectively. The chemical identities of the separated components were determined by matching their recorded mass spectra with the data bank mass spectra (general purpose, terpene ThermoQuest and NIST libraries) provided by the instrument software and by comparing their calculated Kovats retention indices with literature values measured on columns with identical polarity.

GC-FID was also carried out to obtain complementary results of those obtained by GC-MS. GC-FID was performed on a ThermoQuest gas chromatograph coupled to FID (QP2010) equipped with HP-5 capillary column stationary phases. The temperature and splitting ratio were carried out with the same conditions mentioned in GC-MS analysis. Helium (He) was the carrier gas at a flow rate of 1 mL/min.

Identification of the essential oil constituents was accomplished relying on their retention indices on HP-5 column and by computerized matching of the acquired mass spectra with those recorded by Adams [42]. Also, the mass chart of each component matched with MS libraries including Mainlib, Wiley, and Replib. Furthermore, Kovats retention indices were used for identification of the essential oil composition [42].

### 2.3. Oil Extraction

Leaves of* A. judaica* were collected from plants before blooming during spring 2011 grown at Al-Mudawarah area, Ma'an governorate, Jordan. The collected material was identified by Professor Dawoud Al-Eisawi, Plant Taxonomist, Department of Biology, Faculty of Science, The University of Jordan. Voucher specimen (AJ-I 2011) was deposited at the Herbarium of the National Center for Agricultural Research and Extension in Jordan.

The oil of air-dried and finely ground leaves of* A. judaica* was obtained by hydrodistillation using a Clevenger-type apparatus. Distillation was performed using 100 g of dried plant material in 2.5 L distilled water for 3 h at the Pharmaceutical Sciences Department Lab, Pharmacy Faculty, The University of Jordan. Three replicates were carried out. The oil was obtained pure and clearly separated from water layer. It was dried over anhydrous sodium sulfate and stored in a dark glass bottle at 4°C until analysis. The yield of oil was 0.50% ± 0.04% based on the dried weight of sample.

### 2.4. Evaluation of Degassing Effect of* A. judaica* Oil on CO_2_ Saturated Solution by FT/ATR-IR Analysis

Saturated solution of CO_2_ prepared by passing CO_2_ gas in distilled water was incubated at 0°C and then sonicated to remove the suspended bubbles of gas. Equal volumes (9 mL each) were used for blank, test, positive, and negative control solutions. A drop from CO_2_ solution was added to the sample lens of attenuated total reflectance of IR spectrometer and considered as blank solution. The test solution was prepared by adding 1 mL of methanolic solution of* A. judaica* oil (10 mg/mL) to 9 mL of CO_2_ solution. Negative control was prepared by adding 1 mL of methanolic solution of paraffin oil (10 mg/mL) to 9 mL of CO_2_ solution whereas positive control was prepared by addition of 1 mL of methanolic solution of thymol (10 mg/mL) to 9 mL of CO_2_ solution. IR spectrum and the corresponding first derivative were obtained from FT/ATR-IR instrument. First derivative of signal characteristic of asymmetric carbonyl stretching of CO_2_ was measured and compared with negative and positive controls.

Validation of the FT/ATR-IR method has been fulfilled by studying linearity, accuracy, limit of detection (LOD), limit of quantification (LOQ), precision, selectivity, and robustness.

#### 2.4.1. Linearity

Eight different concentrations of* A. judaica* oil and another set of eight solutions of thymol in the range of 0–2.0 mg/mL were prepared for linearity study and measured in triplicate by FT/ATR-IR system. Signal absorbance values were considered and then calibration curve was plotted.

#### 2.4.2. Accuracy, LOQ, and LOD

Accuracy was evaluated by comparing the results of IR analysis with results of acid-base titration method carried out by titrating 10 mL of each solution prepared in linearity study against NaOH (0.01 N). Phenolphthalein was used as indicator. The least concentration of carbon dioxide solution treated with the oil and depicted linearity with the higher concentrations was considered as limit of quantification (LOQ). The exact quantity of carbon dioxide at limit of quantification was determined by the titration method described previously. Limit of detection (LOD) was considered as the least concentration below which IR was not able to differentiate between signal and noise and the response kept constant and independent of carbon dioxide concentration. The exact amount of carbon dioxide content at limit of detection was also determined by the titration method.

#### 2.4.3. Precision

The precision study expresses degree of agreement among results when the method is applied similarly to multiple homologous samples. Inter- and intraday precisions were evaluated by calculating the percentage of relative standard deviation (%RSD). Six samples at a concentration of 0.5 mg/mL of* A. judaica* oil and another set of six samples containing 0.5 mg/mL of thymol were prepared to evaluate the precision of the method. Each solution was tested in triplicate.

#### 2.4.4. Selectivity and Robustness

Selectivity of the method was evaluated by reading samples containing* A. judaica* oil and others containing thymol after substituting the saturated carbon dioxide solution with water free of carbon dioxide. On the other hand, robustness was evaluated by measuring the response of carbon dioxide signal of stock solution of carbon dioxide kept at 25°C. And then it was changed deliberately within a deviation of ±2°C to study the effect of a slight temperature variation on the carbon dioxide signal. After each IR scan of solutions, droplets were left for 30 min to study the effect of time on the carbon dioxide content.

### 2.5. Evaluation of Surface Activity of* A. judaica* Oil on D_2_O/CDCl_3_ Interface by ^1^H-NMR Analysis

35 mL of deuterated water (D_2_O contains 0.1% H_2_O) was added to 0.6 mL deuterated chloroform (CDCl_3_, 1% TMS) containing 10 mg of* A. judaica* oil. ^1^H-NMR was scanned from the chloroform layer. Solubilized content of water in chloroform layer due to surface activity of the essential oil was measured by integration of H_2_O signal at chemical shift of 4.8 ppm and TMS signal at zero points. The result was compared with blank solution prepared with the same manner without use of* A. judaica* oil. The obtained percentage of water in chloroform layer was compared with triplicate results of titration by Karl Fischer reagent using absolute methanol as a solvent.

## 3. Results and Discussion

### 3.1. Evaluation of Degassing Effect of* A. judaica* Oil on CO_2_ Saturated Solution by FT/ATR-IR Analysis

**Figure 1:**
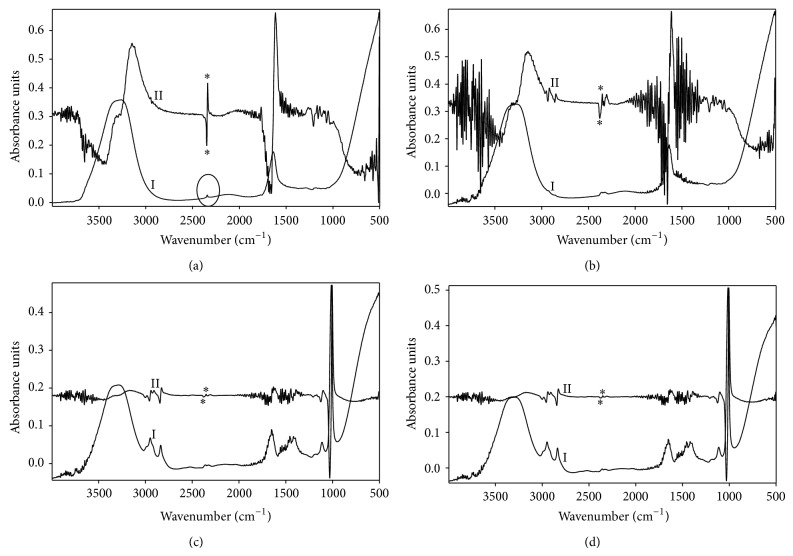
IR and its first derivative analysis for blank CO_2_ solution (a), CO_2_ solution treated with paraffin/methanol as negative control (b), CO_2_ solution treated with* A. judaica* oil/methanol as tested solution (c), and CO_2_ solution treated with thymol/methanol as positive control (d).* A. judaica* reduces the altitude of first derivative signal of CO_2_ stretching showing almost the absorbance of equal amount of thymol.

Carbon dioxide is one of the major gas constituents generated by microflora in the gut lumen [43]. Therefore, the study of antifoaming activity of* A. judaica* oil against CO_2_ bubble formation was undertaken. Carbon dioxide has a characteristic IR signal at 2350 cm^−1^ resulting from asymmetric stretching of carbonyl groups of the molecule (Figure 1) [44]. This peak, predominately, appears in a region that either is usually clear of signals or contains weak peaks [45]. Although the contribution of atmospheric CO_2_ is trivial relative to the sample CO_2_, background spectrum was subtracted from each sample.

Since baseline drift and interference with carbonyl stretching signal limit IR suitability for quantitative analysis, the first derivative of the spectra was employed. Mathematical derivatization minimizes the baseline drift and anomaly of the spectrum [46–48]. And also it enhances the selectivity of the analysis due to the improvement of the peak shape and resolution [46]. The absorbance of the first derivative signal, corresponding to carbonyl stretching, was attributed to CO_2_ content in the solution. The blank sample depicted an absorbance of 0.22 absorbance units for the characteristic signal of CO_2_ stretching (Figure 1(a)). Paraffin oil, which lacks hydrophilic-lipophilic balance and, consequently, depicts very weak antifoaming activity, was chosen as a negative control. However, it was necessary to add cosolvent such as methanol to dissolve paraffin oil in aqueous solution of CO_2_. As shown in Figure 1(b), 50% reduction of the absorbance was observed. The observed reduction is equivalent to methanol standard rather than paraffin oil in the sample (data not shown). Interestingly, CO_2_ content in the sample treated with* A. judaica* essential oil was dramatically reduced to less than 5% relative to the blank sample (Figure 1(c)), representing a strong antifoaming activity of the essential oil. The CO_2_ content in oil-treated sample is quite low and almost identical to CO_2_ content in a solution containing thymol standard (Figure 1(d)). Because of its phenolic group, which provides substantial balance between hydrophilicity and lipophilicity, thymol has a strong surface activity in comparison with other essential oils [23, 24].

The antifoaming activity of* A. judaica* is expected to be directly related to the coalescence of small bubbles of CO_2_ gas. This should generate unstable large bubbles that are removed easily and quickly from the aqueous solution. The proposed mechanism is speculated to take place inside GI tract and is likely responsible for the carminative effect of* A. judaica* against abdominal flatulence.

**Figure 2:**
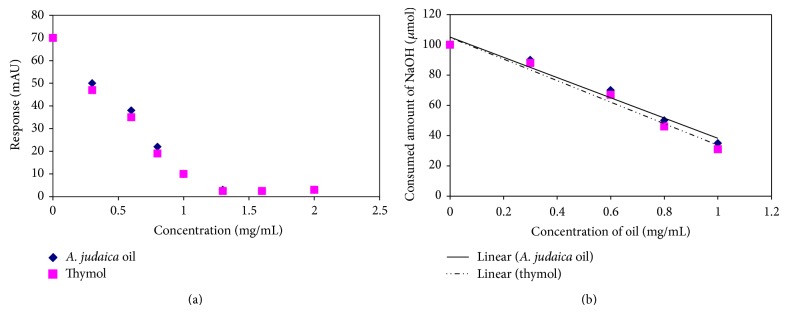
(a) Linearity study for the effect of* A. judaica* oil and thymol on the response of IR carbonyl stretching of CO_2_; samples of concentrations within the range of 0-1.0 mg/mL showed a linear behavior (*R* > 0.99). (b) Linearity study of CO_2_ solution treated with several concentrations of* A. judaica* oil and thymol versus the consumed amount of NaOH needed to neutralize them (*R* > 0.98). The close similarity between slopes of curves (a) and (b) implies the accuracy of IR method in quantifying CO_2_ content.

The validation protocol of FT/ATR-IR method was carried out according to the procedure mentioned in Section 2.4. As depicted in Figure 2, there is a linear relationship between the concentration of* A. judaica* oil and the absorbance value of IR signal of carbon dioxide within the range of 0-1.0 mg/mL. The regression coefficient of the five samples having concentration values within the linear range exceeded 0.99. Additionally, thymol as a positive control almost behaved similar to* A. judaica* oil. The point at which the least response of carbon dioxide signal is linear with higher concentration of carbon dioxide solutions was considered as limit of quantification (LOQ). As observed from Figure 2, LOQ for both* A. judaica* oil and thymol was found for carbon dioxide solution containing 1 mg/mL of either* A. judaica* oil or thymol. Titration (mentioned in Section 2.4 of large volumes of these solutions) gave rise that the drop (0.05 mL) of solution contained 8 *μ*g CO_2_. Thus LOQ of carbon dioxide by IR method is 160 *μ*g/mL. On the other hand, limit of detection (LOD) was considered as the least concentration below which IR was not able to differentiate between signal and noise. This occurred at a concentration of 1.3 mg/mL of either* A. judaica* oil or thymol. Titration method revealed that LOD was 50 *μ*g/mL. Accuracy of IR method was tested by comparing the slope of CO_2_ signal declination (Figure 2) with slope of linear curve representing consumed volumes of 0.01 N NaOH required to neutralize CO_2_ solutions. As depicted in Figure 2, the slope of curve A is almost identical to curve B and is found to be around 65. This implied that the IR signal represents accurately the content of CO_2_ gas in solutions. Precision was tested by adopting the relative standard deviations (RSD) of inter- and intraday variability which was found to be less than 5.0% for the samples mentioned in Section 2.4. Samples of* A. judaica* oil and others of thymol solutions which are free of CO_2_ represented charts free of any signal at the wavenumber of CO_2_ stretching. This emphasizes the selectivity of the method for carbon dioxide. Moreover, robustness of the method was tested by studying temperature effect with a deviation of ±2°C from the room temperature and time effect for 30 min which were studied according to the procedure in Section 2.4. Deviations of results in both cases were below 5% of the original values of signal absorbances.

### 3.2. Evaluation of Surface Activity of* A. judaica* Essential Oil on D_2_O/CDCl_3_ Interface by ^1^H-NMR Analysis

**Figure 3:**
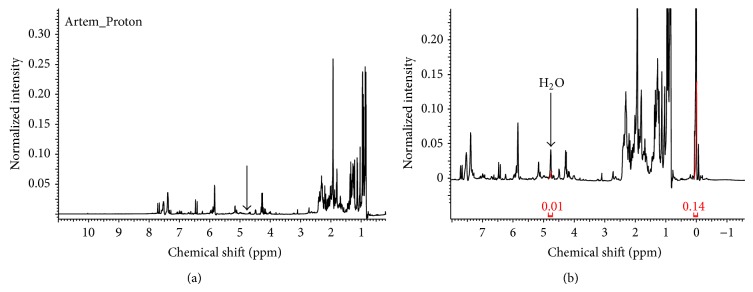
^1^H-NMR spectra of CDCl_3_ layer mixed with few drops of D_2_O (a) and the same sample treated with 10 mg of* A. judaica* oil (b).* A. judaica* oil was able to solubilize water in chloroform layer due its surface activity.

The antifoaming activity of* A. judaica* essential oil can be probably linked to its surface activity. To test the ability of* A. judaica* essential oil to reduce the interfacial tension between water and chloroform phases, ^1^H-NMR was utilized as a quantitative tool of analysis. According to our findings, while a peak corresponding to water was absent in the NMR spectrum of CDCl_3_ layer, as shown in Figure 3(a), a characteristic peak of water resonance at 4.8 ppm after the addition of* A. judaica* oil was detected (Figure 3(b)). The solubilized water content in chloroform is a direct proof of the oil's surface activity, which reduces the interfacial tension between the aqueous and oil layers. Therefore, we hypothesize that the antifoaming character of the oil, which was observed by IR, might be directly related to the surface activity demonstrated by NMR. Based on (1), the percentage of water solubilized in chloroform layer by the oil was determined to be approximately 4%. This result was also confirmed by titration with Karl Fischer reagent which provided a close result to NMR model. Triplicate samples contained water percentage of 3.8 ± 0.3%:

(1) $$\begin{array}{c} P_{W}=\frac{\int W}{\int T}\times \frac{H_{T}{C_{T}D}_{T}M_{W}F100\%}{H_{W}M_{T}D_{C}}, \end{array}$$

where *P*_*W*_ is the percentage of water in chloroform layer, ∫*W* is integration of H_2_O peak at 4.8 ppm, ∫*T* is integration of TMS peak at zero points, *H*_*T*_ is number of hydrogen atoms in the molecule of TMS (12), *H*_*W*_ is the number of hydrogen atoms in the molecule of H_2_O (2), *C*_*T*_ is concentration of TMS in chloroform (1% v/v), *D*_*T*_ is density of TMS (0.648 g/mL), *M*_*W*_ is molecular weight of H_2_O (18 g/mole), *M*_*T*_ is molecular weight of TMS (88 g/mole), *F* is purity factor of D_2_O (99.9%), and *D*_*C*_ is density of chloroform (1.483 g/mL).

### 3.3. Composition of* A. judaica* Essential Oil by Using GC-MS and GC-FID

The essential oil extracted from* A. judaica* possessed a strong greenish color with aromatic smell. The identified compounds are listed in Table 1. The essential oil was obtained in a yield of 0.50% on a dry weight basis. According to GC-MS and GC-FID analysis, the isolated oil contains considerable content of octenyl acetate (2E) (26.6%), p-cymen-8-ol (26.6%), and p-menth-3-en-8-ol (21.6%) (Table 1). The second major constituents were pentylcyclohexa-1,3-diene (6.6%), verbanyl acetate (4.4%), isophorone (3.5%), and artemisia ketone (3.5%). The remaining constituents were found in trace amounts less than 0.1%. Certain constituents such as 1,8-cineole, *α*-thujone, camphor, thymol, lavandulol, and santolina triene were reported in previous studies [49], while no evident existence of these components appeared in our sample. Structural analysis revealed that the oil comprises oxygenated terpenes including alcoholic, ketonic, and esteric compounds. Octenyl acetate (26.6%) and p-menth-3-en-8-ol (21.6%) were the major esteric and alcoholic constituents, respectively. These oxygenated compounds possess hydrophilic oxygenated part and hydrophobic terpene part. This provides the molecules with an optimum surface activity against interfacial tension [35, 50, 51]. According to our results, we propose that oxygenated terpenes are responsible for the surface activity of* A. judaica* oil and, consequently, its carminative effect.

**Figure 4:**
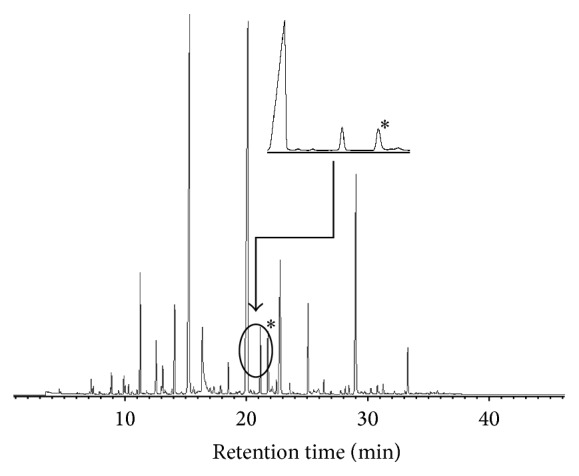
GC-MS chromatogram of* A. judaica* oil. (∗) peak appeared as single peak even in diluted samples.

**Figure 5:**
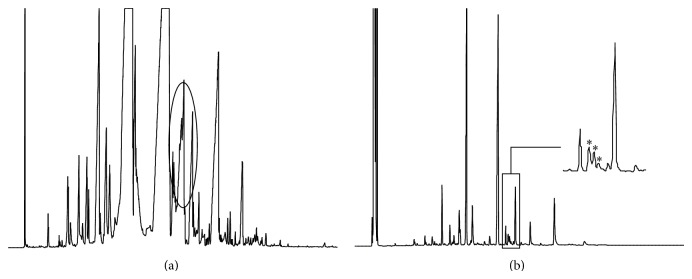
GC-FID chromatogram of* A. judaica* oil. (∗) peaks appeared as three peaks depicting the ability of FID detector in conserving the chromatographic separation in comparison with MS detector which possesses relatively higher dead volume.

Interestingly, comparing the chromatogram obtained from GC-MS (Figure 4) with the one obtained from GC-FID (Figure 5), it was noticed that better resolution was attained by GC-FID. For example, the peak corresponding to the Kovats index of p-cymen-8-ol (Figure 4) depicted three relevant peaks on the chromatogram of GC-FID (Figure 5). This might be due to the difference in dead volume of the detectors. Whereas FID has a compacted geometry and consequently a limited dead volume, MS detector has a relatively large dead volume due to its ionizer, analyser, and interface spaces [36]. Hence, the chromatographic separation of GC-MS might be partially reduced due to the relatively large dead volume of MS detector.

The analysis of GC-FID confirmed the presence of related isomers as shown in Figure 5. Further work was undertaken to identify the detected subpeaks according to the method described by Adams for the identification of essential oil components using GC and MS [42]. Characteristic fragments of p-cymen-8-ol isomers including 43, 65, 91, 135, and 150 m/z were identified for assigned chromatographic peaks. Thus, the assigned peaks (Figure 5) are speculated to be p-cymenol isomers including p-cymen-8-ol, p-cymen-3-ol, and p-cymen-5-ol. To the best of our knowledge, this is the first report in literature providing an evidence for the presence of p-cymen-3-ol and p-cymen-5-ol in* A. judaica*. However, further analysis using standard of p-cymenol isomers should confirm the presence of them.

Conclusively, FT/ATR-IR method was found valid in quantifying the content of carbon dioxide in the sample through applying first derivatization to the carbonyl signal. The intensity of the signal and the steady baseline are factors responsible for the suitability of the method for quantitative analysis. This method showed that* A. judaica* oil effectively reduced the response of CO_2_ signal equivalent to thymol standard. The oil ability in reducing carbon dioxide content is related to its activity against interfacial tension which was studied by ^1^H-NMR spectroscopy in a D_2_O/CDCl_3_ system. This can explain the carminative activity of* A. judaica* oil which is expected to remove gases in the stomach body to stomach fundus where gases are collected and then removed externally.* A. judaica* oil might also reduce formation of gases in intestines through relaxation of intestinal muscles. GC-MS and GC-FID structural analysis revealed that* A. judaica* oil consists of oxygenated terpenes having activity against interfacial tension.

## Figures and Tables

**Table 1:** Chemical composition of *A. judaica* essential oil from Al-Mudawarah area in Jordan. The oil contains several oxygenated terpenes possessing activity against interfacial tension.

Chemical compound	Actual KI	Theoretical KI	% of content	Chemical compound	Actual KI	Theoretical KI	% of content
*β*-Citronellene	945	950	0.8	Octenyl acetate (2E)	1208	1213	26.6
Linalool oxide (dehydro,trans)	996	993	<0.1	Carveol (cis)	1228	1229	0.9
Limonene	1028	1029	<0.1	Carvenone	1262	1258	<0.1
*β*-Ocimene (cis)	1034	1039	<0.1	Limonene-10-ol	1287	1289	<0.1
Artemisia ketone	1058	1062	3.5	*β*-Elsholtzia ketone (dehydro)	1302	1302	<0.1
Artemisia alcohol	1071	1083	<0.1	Cryptone (4-hydroxy)	1318	1315	<0.1
m-Cymenene	1091	1085	<0.1	Verbanyl acetate	1325	1321	4.4
Phenylpropanal (2)	1104	1102	<0.1	Piperityl acetate (trans)	1345	1346	<0.1
Maltol	1109	1108	<0.1	Ethyl cinnamate (Z)	1380	1377	3.3
*β*-Thujone	1121	1114	<0.1	*α*-Barbatene	1412	1407	<0.1
isophorone	1127	1121	3.5	Decenal (4E), diethyl acetal	1476	1475	0.3
p-Menth-3-en-8-ol	1153	1150	21.6	Dihydro-β-agarofuran	1506	1503	<0.1
Pentyl cyclohexa-1,3-diene	1163	1160	6.6	Bazzanene	1520	1520	0.4
p-Cymen-8-ol	1179	1180	26.6	Laciniata furanone	1532	1532	<0.1
2-Allyl phenol	1194	1193	<0.1	*β*-Copaen-4-*α*-ol	1583	1590	1.6
Verbenone	1201	1205	2.0				

## References

[1] Alzweiri M., Sarhan A. A., Mansi K., Hudaib M., Aburjai T. (2011). Ethnopharmacological survey of medicinal herbs in Jordan, the Northern Badia region. *Journal of Ethnopharmacology*.

[2] Charchari S. (2002). The essential oil of *Artemisia judaica* L. from Algeria. *Journal of Essential Oil Research*.

[3] Fontaine P., Wong V., Williams T. J., Garcia C., Adams J. D. (2013). Chemical composition and antinociceptive activity of California sagebrush (Artemisia californica). *Journal of Pharmacognosy and Phytotherapy*.

[4] Aziz M., Karim A., El-Ouariachi E. M. (2012). Relaxant effect of essential oil of *Artemisia herba-alba* Asso. on rodent jejunum contractions. *Scientia Pharmaceutica*.

[5] Shah A. J., Gilani A.-H., Abbas K., Rasheed M., Ahmed A., Ahmad V. U. (2011). Studies on the chemical composition and possible mechanisms underlying the antispasmodic and bronchodilatory activities of the essential oil of *Artemisia maritima* L. *Archives of Pharmacal Research*.

[6] Shams M., Zeraati F., Araghchian M., Sadeghzadeh S., Torabian S., Razzaghi K. (2012). Topical anti-nociceptive effect of *Artemisia absinthium* extract in male mice. *Acta Horticulturae*.

[7] Abd-Elhady H. (2012). Insecticidal activity and chemical composition of essential oil from *artemisia judaica* L. against *callosobruchus maculatus* (F.) (Coleoptera: Bruchidae). *Journal of Plant Protection Research*.

[8] Badr A., Abo El-Khier Z., Hegazi G. A., Abd El-Kawi A., El-Sawy A. (2012). Genetic variation in seven natural populations of *Artemisia judaica* L. in South Sinai using RAPD markers. *World Applied Sciences Journal*.

[9] Badr A., El-Shazly H. H., Helail N. S., El Ghanim W. (2012). Genetic diversity of Artemisia populations in central and north Saudi Arabia based on morphological variation and RAPD polymorphism. *Plant Systematics and Evolution*.

[10] Zihlif M., Afifi F., Muhtaseb R., Al-Khatib S., Abaza I., Naffa R. (2012). Screening the antiangiogenic activity of medicinal plants grown and sold in Jordan. *Planta Medica*.

[11] Abdelgaleil S. A. M., Abbassy M. A., Belal A.-S. H., Abdel Rasoul M. A. A. (2008). Bioactivity of two major constituents isolated from the essential oil of *Artemisia judaica* L.. *Bioresource Technology*.

[12] Bora K. S., Sharma A. (2011). The genus artemisia: a comprehensive review. *Pharmaceutical Biology*.

[13] Hashem M. (2011). Antifungal properties of crude extracts of five Egyptian medicinal plants against dermatophytes and emerging fungi. *Mycopathologia*.

[14] El-Sharabasy H. M. (2010). Acaricidal activities of *Artemisia judaica* L. extracts against *Tetranychus urticae* Koch and its predator *Phytoseiulus persimilis* athias henriot (Tetranychidae: Phytoseiidae). *Journal of Biopesticides*.

[15] Bilia A. R., Flamini G., Taglioli V., Morelli I., Vincieri F. F. (2002). GC-MS analysis of essential oil of some commercial Fennel teas. *Food Chemistry*.

[16] Gali-Muhtasib H., Mahmud T. H. K., Arjumand A. (2006). Anticancer and medicinal properties of essential oil and extracts of East Mediterranean sage (salvia triloba). *Advances in Phytomedicine*.

[17] Sadraei H., Asghari G. R., Hajhashemi V., Kolagar A., Ebrahimi M. (2001). Spasmolytic activity of essential oil and various extracts of *Ferula gummosa* Boiss. on ileum contractions. *Phytomedicine*.

[18] Cheang W. S., Lam M. Y., Wong W. T. (2013). Menthol relaxes rat aortae, mesenteric and coronary arteries by inhibiting calcium influx. *European Journal of Pharmacology*.

[19] Gaudioso C., Hao J., Martin-Eauclaire M.-F., Gabriac M., Delmas P. (2012). Menthol pain relief through cumulative inactivation of voltage-gated sodium channels. *Pain*.

[20] Gilani A. H., Shah A. J., Zubair A. (2009). Chemical composition and mechanisms underlying the spasmolytic and bronchodilatory properties of the essential oil of *Nepeta cataria* L.. *Journal of Ethnopharmacology*.

[21] Swandulla D., Schafer K., Lux H. D. (1986). Calcium channel current inactivation is selectively modulated by menthol. *Neuroscience Letters*.

[22] dos Anjos J. L. V., Alonso A. (2008). Terpenes increase the partitioning and molecular dynamics of an amphipathic spin label in stratum corneum membranes. *International Journal of Pharmaceutics*.

[23] Sánchez M. E., Turina A. D. V., García D. A., Nolan M. V., Perillo M. A. (2004). Surface activity of thymol: implications for an eventual pharmacological activity. *Colloids and Surfaces B: Biointerfaces*.

[24] Turina A. D. V., Nolan M. V., Zygadlo J. A., Perillo M. A. (2006). Natural terpenes: self-assembly and membrane partitioning. *Biophysical Chemistry*.

[25] Grigoleit H. G., Grigoleit P. (2005). Pharmacology and preclinical pharmacokinetics of peppermint oil. *Phytomedicine*.

[26] Stojanović-Radić Z., Čomić L., Radulović N. (2012). Antistaphylococcal activity of *Inula helenium* L. root essential oil: eudesmane sesquiterpene lactones induce cell membrane damage. *European Journal of Clinical Microbiology and Infectious Diseases*.

[27] Zuzarte M., Vale-Silva L., Gonçalves M. J. (2012). Antifungal activity of phenolic-rich *Lavandula multifida* L. essential oil. *European Journal of Clinical Microbiology and Infectious Diseases*.

[28] Cavanagh H. M. A., Wilkinson J. M. (2002). Biological activities of lavender essential oil. *Phytotherapy Research*.

[29] Nickavar B., Mojab F., Dolat-Abadi R. (2005). Composition of the volatile oil of Thymus daenensis Celak. subsp. daenensis. *Journal of Medicinal Plants*.

[30] Danzl D. F. (1992). Flatology. *The Journal of Emergency Medicine*.

[31] Harries N., James K. C., Pugh W. K. (1978). Antifoaming and carminative actions of volatile oils. *Journal of Clinical Pharmacy*.

[32] Joshi K. S., Baumann A., Jeelani S. A. K., Blickenstorfer C., Naegeli I., Windhab E. J. (2009). Mechanism of bubble coalescence induced by surfactant covered antifoam particles. *Journal of Colloid and Interface Science*.

[33] Sivaramakrishnan C. N. (2004). Foam control agents. *Colourage*.

[34] Sivaramakrishnan C. N. (2007). Silicone surfactants. *Colourage*.

[35] Manabe A., Nakayama S., Sakamoto K. (1987). Effects of essential oils on erythrocytes and hepatocytes from rats and dipalmitoyl phosphatidylcholine-liposomes. *The Japanese Journal of Pharmacology*.

[36] Alzweiri M., Watson D. G., Parkinson J. A. (2013). Metabonomics as a clinical tool of analysis: LC-MS approaches. *Journal of Liquid Chromatography and Related Technologies*.

[37] Sidhu H. K., Haagenson D. M., Wiesenborn D. P. Nondestructive analysis of single plant canola (*Brassica napus*) seeds using near infra-red spectroscopy.

[38] Wen J.-L., Xue B.-L., Xu F., Sun R.-C. Quantitative structures and thermal properties of the birch lignins during ionic liquid-based biorefinery.

[39] Fra̧ckowiak A., Kokot Z. J. (2012). Quantitative analysis of nofrloxacin by ^1^H NMR and HPLC. *Acta Poloniae Pharmaceutica—Drug Research*.

[40] Verma S. K., Deb M. K. (2007). Direct and rapid determination of sulphate in environmental samples with diffuse reflectance Fourier transform infrared spectroscopy using KBr substrate. *Talanta*.

[41] Zou P., Tu P., Jiang Y. (2013). A simple and specific quantitative method for determination of dictamnine in Dictamni Cortex by ^1^H NMR spectroscopy. *Analytical Methods*.

[42] Adams R. P. (2007). *Identification of Essential Oil Components by Gas Chromatography/Mass Spectrometry*.

[43] Kurbel S., Kurbel B., Včev A. (2006). Intestinal gases and flatulence: possible causes of occurrence. *Medical Hypotheses*.

[44] Sung K., Brown L. R., Toth R. A., Crawford T. J. (2009). Fourier transform infrared spectroscopy measurements of H_2_O-broadened half-widths of CO_2_ at 4.3 *μ*m. *Canadian Journal of Physics*.

[45] Wang T.-H., Craciun R., Dixon D. A., Gole J. L. Induced infrared spectral enhancement in doped TiO_2_.

[46] Li N., Li X.-Y., Zou Z.-X., Lin L.-R., Li Y.-Q. (2011). A novel baseline-correction method for standard addition based derivative spectra and its application to quantitative analysis of benzo(a)pyrene in vegetable oil samples. *Analyst*.

[47] Patel M., Patel B., Parmar S. (2013). Simultaneous estimation of ibuprofen and phenylephrine hydrochloride in bulk and combined dosage form by first derivative UV spectrophotometry method. *Journal of Spectroscopy*.

[48] Wagh D. D., Deshmukh V. V., Vassa S. P., Jain H. K. (2013). Development and validation of first order derivative UV spectrophotometric method for simultaneous estimation of propranolol hydrochloride and flunarizine dihydrochloride in bulk and combined dosage form. *International Research Journal of Pharmacy*.

[49] Dob T., Chelghoum C. (2006). Chemical composition of the essential oil of *Artemisia judaica* L. from Algeria. *Flavour and Fragrance Journal*.

[50] Doll K. M., Moser B. R., Erhan S. Z. (2007). Surface tension studies of alkyl esters and epoxidized alkyl esters relevant to oleochemically based fuel additives. *Energy & Fuels*.

[51] Juven B. J., Kanner J., Schved F., Weisslowicz H. (1994). Factors that interact with the antibacterial action of thyme essential oil and its active constituents. *Journal of Applied Bacteriology*.

